# Characteristics and predictors of persistent symptoms post-COVID-19 in children and young people: a large community cross-sectional study in England

**DOI:** 10.1136/archdischild-2022-325152

**Published:** 2023-03-02

**Authors:** Christina J Atchison, Matthew Whitaker, Christl A Donnelly, Marc Chadeau-Hyam, Steven Riley, Ara Darzi, Deborah Ashby, Wendy Barclay, Graham S Cooke, Paul Elliott, Helen Ward

**Affiliations:** 1 School of Public Health, Imperial College Healthcare NHS Trust, London, UK; 2 School of Public Health, Imperial College London, London, UK; 3 Department of Statistics, University of Oxford, Oxford, UK; 4 MRC Centre for Global infectious Disease Analysis and Abdul Latif Jameel Institute for Disease and Emergency Analytics, Imperial College London, London, UK; 5 Health Data Research (HDR) UK, Imperial College London, London, UK; 6 Institute of Global Health Innovation, Imperial College London, London, UK; 7 Department of Infectious Disease, Imperial College London, London, UK; 8 National Institute for Health Research Imperial Biomedical Research Centre, Imperial College London, London, UK; 9 MRC Centre for Environment and Health, Imperial College London, London, UK; 10 UK Dementia Research Institute, Imperial College London, London, UK

**Keywords:** Covid-19, Adolescent Health, Epidemiology, Infectious Disease Medicine, Paediatrics

## Abstract

**Objective:**

To estimate the prevalence of, and associated risk factors for, persistent symptoms post-COVID-19 among children aged 5–17 years in England.

**Design:**

Serial cross-sectional study.

**Setting:**

Rounds 10–19 (March 2021 to March 2022) of the REal-time Assessment of Community Transmission-1 study (monthly cross-sectional surveys of random samples of the population in England).

**Study population:**

Children aged 5–17 years in the community.

**Predictors:**

Age, sex, ethnicity, presence of a pre-existing health condition, index of multiple deprivation, COVID-19 vaccination status and dominant UK circulating SARS-CoV-2 variant at time of symptom onset.

**Main outcome measures:**

Prevalence of persistent symptoms, reported as those lasting ≥3 months post-COVID-19.

**Results:**

Overall, 4.4% (95% CI 3.7 to 5.1) of 3173 5–11 year-olds and 13.3% (95% CI 12.5 to 14.1) of 6886 12–17 year-olds with prior symptomatic infection reported at least one symptom lasting ≥3 months post-COVID-19, of whom 13.5% (95% CI 8.4 to 20.9) and 10.9% (95% CI 9.0 to 13.2), respectively, reported their ability to carry out day-to-day activities was reduced ‘a lot’ due to their symptoms. The most common symptoms among participants with persistent symptoms were persistent coughing (27.4%) and headaches (25.4%) in children aged 5–11 years and loss or change of sense of smell (52.2%) and taste (40.7%) in participants aged 12–17 years. Higher age and having a pre-existing health condition were associated with higher odds of reporting persistent symptoms.

**Conclusions:**

One in 23 5–11 year-olds and one in eight 12–17 year-olds post-COVID-19 report persistent symptoms lasting ≥3 months, of which one in nine report a large impact on performing day-to-day activities.

**WHAT IS ALREADY KNOWN ON THIS TOPIC**

- The most common persistent symptoms post-COVID-19 reported across existing studies in children are headaches, fatigue, insomnia and anosmia. However, symptom persistence estimates in children post-COVID-19 vary substantially from 1%–51% due to heterogeneous study designs, variable follow-up periods and differing definitions.

**WHAT THIS STUDY ADDS**

- In England at some point since the start of the pandemic until the end of March 2022, 4.4% of 5–11 year-olds and 13.3% of 12–17 year-olds with prior symptomatic infection had persistent symptoms for 3 months or more following COVID-19. Approximately one in nine of these children reported a large impact in their ability to carry out day-to-day activities due to their symptoms. The most common persistent symptoms were coughing, headaches and loss or change of sense of smell or taste.

**HOW THIS STUDY MIGHT AFFECT RESEARCH, PRACTICE OR POLICY**

- This study shows that persistent symptoms post-COVID-19 are multiple and varied. These findings have implications for researchers, clinicians and affected families in understanding the prevalence and manifestation of long COVID in children and young people.

## Introduction

Compared with adults, children and young people (CYP) are more likely to be asymptomatic or develop mild, transient symptoms following SARS-CoV-2 infection,^1^ and life-threatening illness and death from COVID-19 are rare.^1^ However, like adults, CYP who have been infected with SARS-CoV-2 may experience persistent, postacute symptoms, which may last for many months.^2 3^ Frequently termed ‘long COVID’ or ‘post-COVID-19 condition’, recently published systematic reviews indicate that most studies have been conducted in adults.^3–7^

The most common persistent symptoms post-COVID-19 reported across existing studies in CYP are headaches, fatigue, insomnia and anosmia.^8–13^ However, estimates of symptom persistence vary substantially ranging from 1% to 51%, arguably due to heterogeneous study designs, follow-up periods and definitions.^8–17^ A recent meta-analysis of five controlled studies in CYP found that the frequency of the majority of reported persistent symptoms was similar in SARS-CoV-2 positive cases and SARS-CoV-2 negative controls. Small but significant increases in the pooled risk difference were observed for loss of smell (8%), headaches (5%), cognitive difficulties (3%) and sore throat and eyes (2% each).^6^

Here, we use data from the REal-time Assessment of Community Transmission-1 (REACT-1) study to estimate the prevalence of, and associated risk factors for, persistent symptoms post-COVID-19 among CYP aged 5–17 years in England.

## Methods

### Study design and participants

During each round of the REACT-1 study, a random subset of the population (over 5 years old) of England was chosen from the National Health Service (NHS) general practitioner’s list and invited to participate in the study.^18^ There was a round of the study approximately monthly from May 2020 to March 2022, with each round of data collection including between 15 000 and 35 000 participants aged 5–17 years. The study protocol aimed to achieve approximately equal sample sizes from each lower tier local authority (n=315).^19^ Individuals who agreed to participate provided a self-administered throat and nose swab (parent/guardian administered for those aged 5–12 years old) that underwent PCR testing to determine the presence of SARS-CoV-2. In addition, participants completed an online questionnaire (parent/guardian completed by proxy for those aged 5–12 years old), which included information on demographic variables, recent symptoms, whether or not they thought that they had had COVID-19 and whether or not they had had a previous PCR test. From round 10, participants were asked about persistent symptoms related to previous COVID-19 and severity and duration of these symptoms.^18^ These included a set of 30 clinically relevant symptoms potentially related to COVID-19. The questionnaires used and a table showing the sampling dates and response rates for REACT-1 are available on the study website.^20^

### Data analysis

We estimated the prevalence of persistent symptoms at 3 months postsymptom onset in individuals with a history of COVID-19. We included all self-reported prior COVID-19 episodes including test confirmed and suspected but not confirmed. We only included those who reported onset of COVID-19 ≥3 months prior to the date of the survey and for whom we had complete data. We reported persistent symptoms by age, sex, ethnicity, presence of a pre-existing health condition, index of multiple deprivation (IMD),^21^ COVID-19 vaccination status and dominant UK circulating SARS-CoV-2 variant at time of symptom onset. A valid COVID-19 vaccine dose was defined as a date of vaccination 14 days or more prior to COVID-19. The wild-type strain was dominant in the UK prior to December 2020. Alpha dominated between December 2020 and April 2021 followed by delta (May 2021 to mid-December 2021) and omicron (late December 2021 onwards).^22^

To estimate background prevalence of symptoms, we used data on history of any of 26 symptoms lasting 11 or more days (that were present within the 7 days prior to questionnaire completion) for children aged 5–17 years who had a negative PCR test in the REACT-1 study. The data were weighted (by sex, age, ethnicity, lower tier local authority population and IMD) to take account of the sampling design and differential response rates^18^ to obtain prevalence estimates that were representative of the 5–17 year-old population of England.

We used logistic regression to quantify the associations of age, sex, ethnicity, presence of a pre-existing health condition, IMD, COVID-19 vaccination status and dominant UK circulating SARS-CoV-2 variant at time of symptom onset with persistence of symptoms at 3 months or more. ORs and 95% CIs were estimated.

Methods and results of a supplementary cluster analysis of persistent symptoms are available in online supplemental file 1.

10.1136/archdischild-2022-325152.supp1 Supplementary data

Data were analysed using the statistical package STATA V.15.0.

## Results

Of 191 593 REACT-1 participants aged 5–17 years, 78 948 (41.2%) did not attempt the questionnaire. Of those (or parent/guardian) who completed the questionnaire, 111 444/112 645 (98.9%) reported whether the individual had previously had COVID-19 or not (online supplemental figure S1). Differential non-response was observed by age, sex, ethnicity and deprivation (online supplemental table S1), similar to non-response characteristics of REACT-1 overall.^23^ Of participants reporting a COVID-19 episode, 10 059/22 169 (45.4%) reported a valid date of symptom onset ≥3 months before their survey date, providing our denominator population for estimates of persistent symptoms. Table 1 shows the key characteristics of these participants.

10.1136/archdischild-2022-325152.supp2 Supplementary data

10.1136/archdischild-2022-325152.supp3 Supplementary data

**Figure 1:**
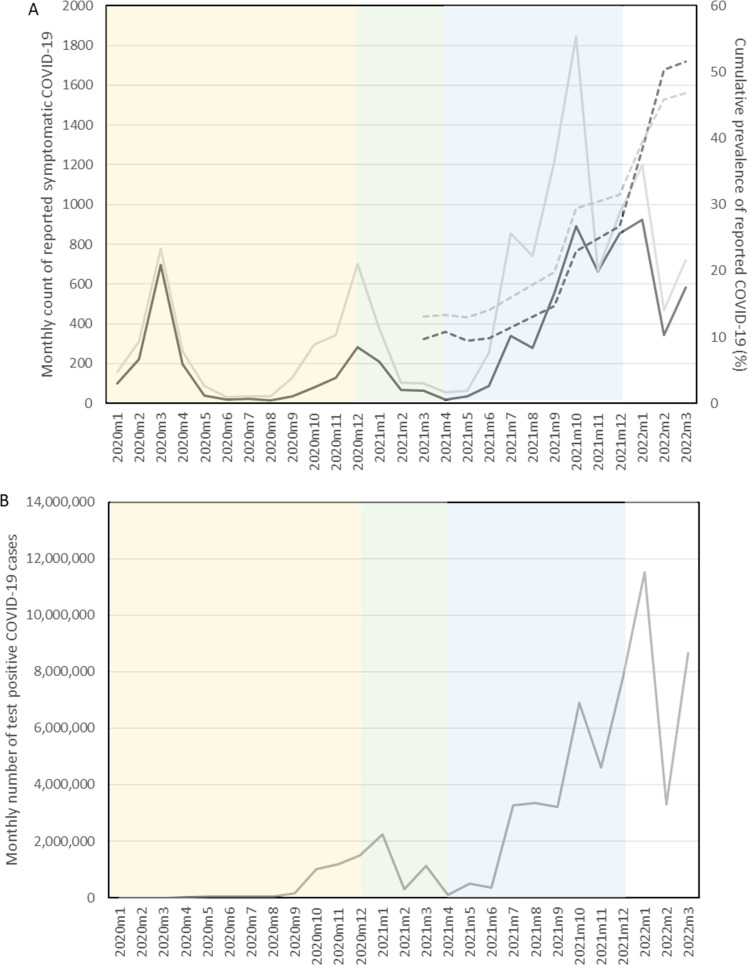
Reconstruction of epidemic curve from study participants with valid date of symptom onset alongside cumulative prevalence of reported COVID-19 and official case numbers for England reported by the UK Government. (A) Number of reported COVID-19 episodes by month^a^ in REACT-1 (solid black line: 5–11 year-olds/sold grey line: 12–17 year-olds) (left y-axis) alongside cumulative prevalence of reported COVID-19 by month in which REACT-1 study round was completed^b^ (dotted black line: 5–11 year-olds/dotted grey line: 12–17 year-olds) (right y-axis). (B) Number of test positive COVID-19 cases by month in England^c^ (solid dark grey line). Data from UK Government website.^28^ Shading (dominant SARS-CoV-2 variant): yellow: wild type; green: alpha; blue: delta; white: omicron. ^a^n=17 585: asymptomatic individuals and symptomatic participants whose date of COVID-19 was unknown are excluded; ^b^n=27 035: individuals reporting a history of COVID-19; ^c^widespread testing for SARS-CoV-2 only became available in the UK from April 2020. REACT-1, REal-time Assessment of Community Transmission-1.

**Table 1:** Numbers and proportions of participants who reported one or more symptoms (from a list of 30 surveyed symptoms) of COVID-19 at 3 months postsymptom onset, among symptomatic participants for whom we have 3-month follow-up and complete data, n=10 059

Category	Participants aged 5–11 years			Participants aged 12–17 years		
	No. reporting one or more symptoms at onset of COVID-19	No. symptomatic at 3 months	% symptomatic at 3 months	No. reporting one or more symptoms at onset of COVID-19	No. symptomatic at 3 months	% symptomatic at 3 months
All participants	3173	138	4.4 (3.7–5.1)	6886	913	13.3 (12.5–14.1)
Sex						
Male	1581	65	4.1 (3.2–5.2)	2885	216	7.5 (6.6–8.5)
Female	1592	73	4.6 (3.7–5.7)	4001	697	17.4 (16.3–18.6)
Ethnicity						
White	2588	122	4.7 (4.0–5.6)	5697	783	13.7 (12.9–14.7)
Mixed	264	9	3.4 (1.8–6.4)	360	41	11.4 (8.5–15.1)
Asian/Asian British	190	3	1.6 (0.5–4.8)	530	51	9.6 (7.4–12.4)
Black/African/Caribbean/black British	41	1	2.4 (0.3–15.7)	126	14	11.1 (6.7–17.9)
Other	35	1	2.9 (0.39–18.1)	81	16	19.8 (12.4–29.9)
IMD quintile						
1 – most deprived	310	10	3.2 (1.7–5.9)	906	144	15.9 (13.7–18.4)
2	497	26	5.2 (3.6–7.6)	1020	158	15.5 (13.4–17.8)
3	643	25	3.9 (2.6–5.7)	1367	169	12.4 (10.7–14.2)
4	744	34	4.6 (3.3–6.3)	1571	188	12.0 (10.5–13.7)
5 – least deprived	979	43	4.4 (3.3–5.9)	2022	254	12.6 (11.2–14.1)
Comorbidities						
No	2701	97	3.6 (3.0–4.4)	5085	544	10.7 (9.9–11.6)
One	412	36	8.7 (6.4–11.9)	1271	231	18.2 (16.1–20.4)
Two or more	54	5	9.3 (3.9–20.5)	513	137	26.7 (23.1–30.7)
Vaccination status at time of infection						
No	3173	138	4.4 (3.7–5.1)	6547	871	13.3 (12.5–14.1)
One dose	0	0	0	310	37	11.9 (8.8–16.0)
At least two doses	0	0	0	29	5	17.2 (7.2–35.7)
Dominant variant at time of infection						
Wild-type (before December 2020)	1722	70	4.1 (3.2–5.1)	2764	309	11.2 (10.1–12.4)
Alpha (December 2020–April 2021)	462	16	3.5 (2.1–5.6)	1032	133	12.9 (11.0–15.1)
Delta (May 2021–December 2021)	989	52	5.3 (4.0–6.8)	3090	471	15.2 (14.0–16.6)

The cumulative prevalence of reported COVID-19 increased from 11.4% (5–11 years: 9.7% and 12–17 years: 13.2%) in Round 10 (March 2021) to 48.2% (5–11 years: 51.6% and 12–17 years: 46.9%) in Round 19 (March 2022). Figure 1 shows how the epidemic in CYP evolved between January 2020 and March 2022.

### Prevalence of persistent symptoms

Overall, 4.4% (95% CI 3.7 to 5.1; 138/3173) of 5–11 year-olds and 13.3% (95% CI 12.5 to 14.1; 913/6886) of 12–17 year-olds symptomatic at infection had one or more persistent symptoms (from a list of 30 surveyed symptoms) for≥3 months (table 1, online supplemental figure S2). Of which, 11.2% (95% CI 9.4 to 13.3) reported their ability to carry out day-to-day activities was reduced “a lot” due to their symptoms (table 2).

**Figure 2:**
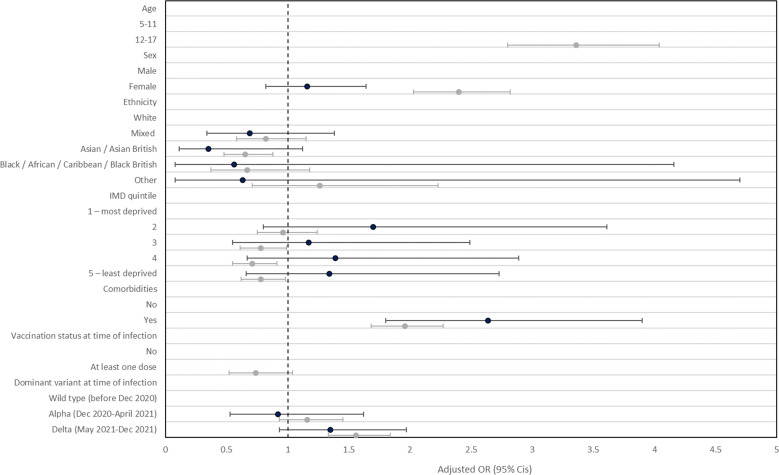
Logistic regression models with one or more symptoms at 3 months (y/n) as the binary outcome variable^a^. adjusted odds ratios (dot) and 95% CIs (line) presented separately for participants aged 5–11 years (black) and 12–17 years (grey), n=10 059. ^a^Mutually adjusted for age, sex, ethnicity, IMD, comorbidities, vaccination status (over 12 year-olds only) and dominant variant at time of infection. No vaccination status estimate provided for children aged 5–11 years because only 5–11 year-olds with certain health conditions, or those with a weakened immune system were being offered vaccination during the study period and no participant aged 5–11 years in our study cohort reported having been vaccinated.

**Table 2:** Key characteristics of persistent symptoms lasting for 3 months or more for symptomatic participants for whom we have 3-month follow-up and complete data, n=10 059

Category	Aged 5–11 years	Aged 12–17 years
	≥3 months n=3173	≥3 months n=6886
Symptom duration	n (%; 95% CI)*	n (%; 95% CI)*
Less than 4 weeks	2797 (88.2; 87.1 to 89.3)	5290 (76.9; 75.8 to 77.8)
4 weeks up to 2 months	183 (5.8; 5.0 to 6.6)	479 (7.0; 6.4 to 7.6)
2 months up to 3 months	52 (1.6; –1.3 to 2.1)	201 (2.9; 2.5 to 3.3)
3 months or more	138 (4.4; 3.7 to 5.1)	913 (13.3; 12.5 to 14.1)
No. of symptoms (at 3 months post-COVID-19)		
No symptoms	3035 (95.7; 94.9 to 96.3)	5973 (86.7; 85.9 to 87.5)
One	55 (1.7; 1.3 to 2.3)	311 (4.5; 4.1 to 5.0)
Two	31 (1.0; 0.7 to 1.4)	272 (4.0; 3.5 to 4.4)
Three	9 (0.3; 0.2 to 0.5)	112 (1.6; 1.4 to 2.0)
Four	14 (0.4; 0.3 to 0.7)	68 (1.0; 0.8 to 1.3)
Five or more	29 (0.9; 0.6 to 1.3)	150 (2.2; 1.9 to 2.6)
	**n=138 (those with symptom duration 3 months or more**)	**n=913 (those with symptom duration 3 months or more**)
Symptoms frequency	n (%; 95% CI)*	n (%; 95% CI)*
Every day	34 (28.6; 21.1 to 37.4)	409 (46.9; 43.6 to 50.2)
Most days	34 (28.6; 21.1 to 37.4)	269 (30.9; 27.9 to 34.0)
Come and go	51 (42.9; 34.2 to 52.0)	194 (22.3; 19.6 to 25.1)
Symptoms reduce ability to carry out day-to-day activities		
A lot	16 (13.5; 8.4 to 20.9)	95 (10.9; 9.0 to 13.2)
A little	59 (49.6; 40.6 to 58.6)	361 (41.5; 38.3 to 44.8)
Not at all	41 (34.5; 26.4 to 43.5)	363 (41.7; 38.5 to 45.0)
Don’t know	3 (2.5; 0.8 to 7.6)	51 (5.9; 4.5 to 7.6)
Accessed medical help for symptoms†		
No	42 (35.3; 27.2 to 44.4)	597 (68.5; 65.3 to 71.5)
Pharmacist/by phone (GP‡/NHS 111§)	46 (33.3; 25.9 to 41.7)	204 (22.3; 19.8 to 25.2)
Visited GP/walk-in centre/Accident and Emergency Department /hospital clinic	46 (33.3; 25.9 to 41.7)	149 (16.3; 14.1 to 18.9)
Hospital admission	8 (6.7; 3.4 to 12.9)	5 (0.6; 0.2 to 1.4)
Symptom duration in those with ≥6-month follow-up, persistent symptoms at 3 months and reported no longer having COVID-19 symptoms	**≥6 months** **n=68** n (%; 95% CI)*	**≥6 months** **n=395** n (%; 95% CI)*
3 months up to 6 months	32 (47.1; 35.3 to 59.2)	189 (47.9; 42.9 to 52.8)
6 months or more	36 (52.9; 40.8 to 64.7)	206 (52.2; 47.2 to 57.1)

*Percentages are calculated from non-missing values.

†Participants who sort more than one type of medical help counted for each type accessed.

‡GP: general practitioner, physician in primary care.

§NHS 111: a free-to-call single non-emergency number medical helpline operating in England.

Among 123 468 REACT-1 PCR-negative individuals, average weighted prevalence of any of 26 symptoms lasting 11 or more days was 2.2% (95% CI 2.1 to 2.3) and 2.6% (95% CI 2.5 to 2.7) in those aged 5–11 and 12–17 years, respectively (online supplemental figure S3).

**Figure 3:**
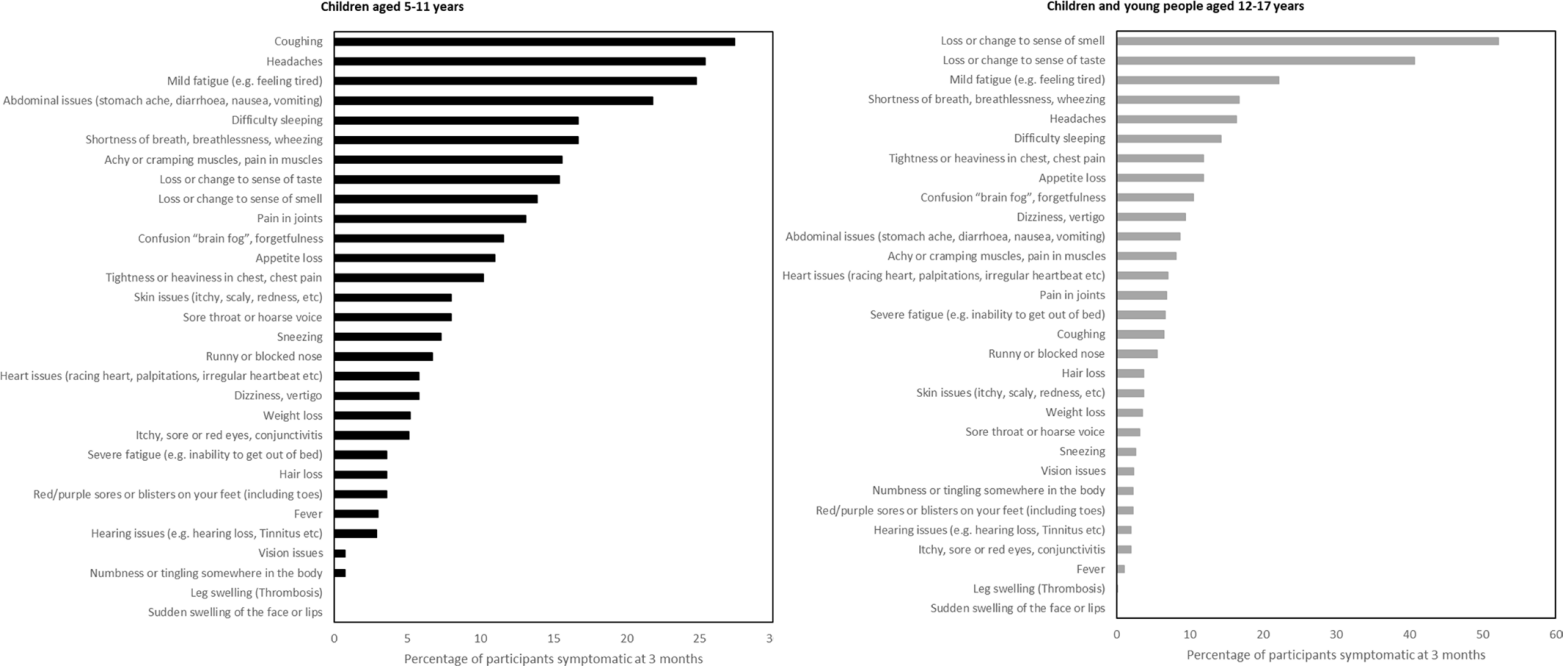
Percentage of individual symptoms among participants with persistent symptoms at 3 months or more post-COVID-19 onset for whom we have 3-month follow-up and complete data, n=1051.

### Factors associated with persistent symptoms

Older children with prior symptomatic infection were three times more likely to report persistent symptoms compared with 5–11 year-olds with prior symptomatic infection, adjusted OR 3.4 (95% CI 2.8 to 4.0) (figure 2). Persistent symptoms post-COVID-19 were higher in those with pre-existing health conditions compared with those without (5–11 years: OR 2.6 (95% CI 1.8 to 3.9); 12–17 years: OR 2.0 (95% CI 1.7 to 2.3)) (figure 2). In 12–17 year-olds, being male, Asian ethnicity and living in more affluent areas were associated with lower odds of persistent symptoms (figure 2). Persistent symptoms in children aged 12–17 years were higher in those infected when delta (OR 1.6; 1.3–1.8) was the dominant SARS-CoV-2 variant circulating in the UK population compared with when the original wild-type strain was dominant earlier in the pandemic (figure 2). With regards to COVID-19 vaccination, persistent symptoms post-COVID-19 were lower in 12–17 year olds with at least one valid vaccine dose compared with those without (OR 0.74 (95% CI 0.52 to 1.04)) (figure 2, online supplemental table S2). However, this was not statistically significant at the 5% level.

### Symptom profiles

The most common symptoms among participants with persistent symptoms were persistent coughing (27.4%; 95% CI 20.5 to 35.6) and headaches (25.4%; 95% CI 18.6 to 33.6) in children aged 5–11 years and loss or change of sense of smell (52.2%; 95% CI 48.9 to 55.5) and taste (40.7%; 95% CI 37.5 to 44.0) in participants aged 12–17 years (figure 3, online supplemental table S3).

## Discussion

In this community-based study in England among children aged 5–17 years with prior COVID-19 and 3 months’ observation time, the prevalence of persistent symptoms for ≥3 months (from a list of 30 clinically relevant symptoms potentially related to COVID-19) was 4.4% and 13.3% in children aged 5–11 years and 12–17 years, respectively. This compares with a background prevalence of symptoms in PCR test negative participants in REACT-1 of 2.2% and 2.6% in 5–11 and 12–17 year-olds, respectively. Our prevalence estimates of persistent symptoms in children post-COVID-19 are therefore approximately twofold and fivefold the background prevalence in children aged 5–11 and 12–17 years, respectively.

This is one of the few studies to provide prevalence estimates of persistent symptoms post-COVID-19 in 5–11 year-olds lasting ≥3 months and to report on how symptoms reduce ability to carry out day-to-day activities and what level of medical care is being accessed for these symptoms. Previous studies in England have focused on older children (CLoCk study: 11–17 years old^9 24^) or on persistent symptoms lasting >28 days (COVID Symptom Study^8^). The COVID Symptom Study found that 4.4% of UK school-aged children (aged 5–17 years) with COVID-19 still reported symptoms at 28 days.^8^ The UK Office for National Statistics (ONS) estimated 7.4% of those aged 2–11 years and 8.2% of those aged 12–16 years still reported symptoms at 12 weeks.^16^ The CLoCk study reported 66.5% of 11–17 year olds who tested SARS-CoV-2 positive had symptoms at 3 months, compared with 53.3% in negative controls.^9 24^ These estimates are much higher than our study. There are several potential explanations for this. First, they limited their study to 11–17 year-olds, among whom persistent symptoms are higher.^6^ Second, participation bias if more CLoCk non-responders had no symptoms compared with REACT-1 participants. This is possible as participants recruited to REACT-1 were not approached with the specific aim of studying persistent symptoms and were offered PCR testing that could have incentivised participation more universally. Third, CLoCk is a prospective longitudinal study, therefore subject to less recall bias. Finally, difference in study period. Our study looked across a longer timeframe during the pandemic with a mixture of the wild-type alpha and delta variants, whereas CLoCk focused on infections during a period when alpha predominated in the UK.^9^ There are differences in the nature of the virus, for example, we know that alpha is more infectious and more likely to be associated with hospitalisations than the original wild-type variant.^25^ This may translate into more cases of long COVID. In addition, the rate of continuing post-COVID-19 symptoms might vary between variants.

Recently, a Delphi research definition for post-COVID-19 condition in CYP has been developed, ‘Post-COVID-19 condition occurs in young people with a history of confirmed SARS-CoV-2 infection, with one or more persisting physical symptoms for a minimum duration of 12 weeks after initial testing that cannot be explained by an alternative diagnosis. The symptoms have an impact on everyday functioning, may continue or develop after COVID-19 infection, and may fluctuate or relapse over time’.^26^ Despite collecting information on how persistent symptoms impact day-to-day activities, we only had information on symptomatic SARS-CoV-2 infections. The research definition of long COVID in CYP includes all confirmed SARS-CoV-2 infection (symptomatic and asymptomatic).^26^ Therefore, our estimates are likely higher and not directly comparable with studies applying this definition.

Few studies have reported on measures of association between key characteristics and persistent symptoms lasting ≥3 months in CYP. We show that increasing age and reporting a pre-existing health condition were associated with higher odds of persistent symptoms following COVID-19, consistent with other studies in children.^9 14^ Persistent symptoms in children aged 12–17 years were higher in those infected during the delta period compared with the original wild-type period earlier in the pandemic. This association is temporal to a large extent as delta was circulating later in the pandemic and therefore may be confounded by the number of previous infections, information on which was not collected as part of our study. A recent case–control study conducted in the UK found a lower odds of long COVID with the omicron versus the delta variant, ranging from OR 0.2 (95% CI 0.2 to 0.3) to 0.5 (95% CI 0.4 to 0.6),^27^ suggesting that the risk of long COVID may vary by variant.

### Strengths and limitations

This study included data from a large random community sample, thus providing representative information on persistent COVID-19 symptoms in children with prior symptomatic infection in the community. The REACT-1 response rate in 5–17 year-olds was low (14.9%), with questionnaire response rate at 6.0%. This was lower than questionnaire response rates of the CloCK and ONS studies, with 11.2% and 12%, respectively.^24^ Similar to these studies, our participants were more likely to be female, older teenagers, white ethnicity, from the southeast (less likely to be from London or the North West) and were more likely to be from the least deprived areas. In contrast to the CloCK study, our participants were broadly more similar to the England population aged 5–17 years, reflecting the random population sampling design^9 24^ (online supplemental table S4).

We used information regarding presence of symptoms rather than whether participants described themselves as having ‘long COVID’ to reduce potential reporting bias. However, the retrospective study design introduces the possibility of recall bias. In addition, we included all self-reported prior COVID-19 episodes including test confirmed and suspected but not confirmed, which introduces the possibility of misclassification bias. When we limited the analysis to test confirmed COVID-19 episodes, the prevalence of persistent symptoms for ≥3 months was higher compared with the prevalence in suspected COVID-19 episodes (5–11 year-olds: 5.1% vs 3.7%, 12–17 year-olds: 16.0% vs 8.6%), and the magnitude and direction of associations between sociodemographic factors and persistent symptoms were similar (online supplemental tables S5 and S6). However, given the limited availability of testing early in the pandemic, it was necessary to include suspected cases. Reassuringly, our epidemic curve produced from participant reported symptom onset date closely tracked the epidemic (figure 1).

A major limitation is the lack of a SARS-CoV-2 negative control group which makes it hard to separate symptoms due to SARS-Co-V2 infection from those caused by normal life, other infections, or the pressures of a pandemic. REACT-1 was originally designed as a surveillance study to provide reliable and timely prevalence estimates of SARS-CoV-2 infection in the community to inform the immediate public health response and so we did not collect baseline information on symptom profiles at the time of symptom onset in those with a history of COVID-19, nor did we collect comparable data on participants with no history of COVID-19. Therefore, our estimates may partly reflect the large list of symptoms we surveyed, many of which are common and not specific to COVID-19. We estimated background prevalence of persistent symptoms to be 2.2% and 2.6% in 5–11 and 12–17 year-olds, respectively. These provide upper bounds for non-COVID-19 related prevalence of persistent symptoms at 3 months or more.

## Conclusion

One in 23 children aged 5–11 years and 1 in 8 individuals aged 12–17 years have persistent symptoms lasting ≥3 months post-COVID-19. Persistent symptoms were multiple and varied with approximately one in nine children reporting a large impact in ability to carry out day-to-day activities due to their symptoms. Our study contributes to a growing evidence base regarding the prevalence, risk factors and characteristics of long COVID in children.

## Data Availability

Data are available on reasonable request. Deidentified individual participant data (including data dictionaries) will be made available, in addition to study protocols, the statistical analysis plan and the informed consent form. The data will be made available on publication to researchers who provide a methodologically sound proposal for use in achieving the goals of the approved proposal. Proposals should be submitted to: react.lc.study@imperial.ac.uk.

## References

[1] Vosoughi F , Makuku R , Tantuoyir MM , et al . A systematic review and meta-analysis of the epidemiological characteristics of COVID-19 in children. BMC Pediatr 2022;22:613. 10.1186/s12887-022-03624-4 36273121 PMC9587668

[2] Sudre CH , Murray B , Varsavsky T , et al . Attributes and predictors of long COVID. Nat Med 2021;27:626–31. 10.1038/s41591-021-01292-y 33692530 PMC7611399

[3] Lopez-Leon S , Wegman-Ostrosky T , Perelman C , et al . More than 50 long-term effects of COVID-19: a systematic review and meta-analysis. Sci Rep 2021;11:16144. 10.1038/s41598-021-95565-8 34373540 PMC8352980

[4] van Kessel SAM , Olde Hartman TC , Lucassen P , et al . Post-acute and long-COVID-19 symptoms in patients with mild diseases: a systematic review. Fam Pract 2022;39:159–67. 10.1093/fampra/cmab076 34268556 PMC8414057

[5] Nasserie T , Hittle M , Goodman SN . Assessment of the frequency and variety of persistent symptoms among patients with COVID-19: a systematic review. JAMA Netw Open 2021;4:e2111417. 10.1001/jamanetworkopen.2021.11417 34037731 PMC8155823

[6] Behnood SA , Shafran R , Bennett SD , et al . Persistent symptoms following SARS-CoV-2 infection amongst children and young people: a meta-analysis of controlled and uncontrolled studies. J Infect 2022;84:158–70. 10.1016/j.jinf.2021.11.011 34813820 PMC8604800

[7] Ludvigsson JF . Case report and systematic review suggest that children may experience similar long-term effects to adults after clinical COVID-19. Acta Paediatr 2021;110:914–21. 10.1111/apa.15673 33205450 PMC7753397

[8] Molteni E , Sudre CH , Canas LS , et al . Illness duration and symptom profile in symptomatic UK school-aged children tested for SARS-CoV-2. Lancet Child Adolesc Health 2021;5:708–18. 10.1016/S2352-4642(21)00198-X 34358472 PMC8443448

[9] Stephenson T , Pinto Pereira SM , Shafran R , et al . Physical and mental health 3 months after SARS-CoV-2 infection (long COVID) among adolescents in England (clock): a national matched cohort study. Lancet Child Adolesc Health 2022;6:230–9. 10.1016/S2352-4642(22)00022-0 35143770 PMC8820961

[10] Say D , Crawford N , McNab S , et al . Post-acute COVID-19 outcomes in children with mild and asymptomatic disease. Lancet Child Adolesc Health 2021;5:e22–3. 10.1016/S2352-4642(21)00124-3 33891880 PMC8057863

[11] Radtke T , Ulyte A , Puhan MA , et al . Long-term symptoms after SARS-CoV-2 infection in school children: population-based cohort with 6-months follow-up. Epidemiology [Preprint]. 10.1101/2021.05.16.21257255

[12] Osmanov IM , Spiridonova E , Bobkova P , et al . Risk factors for long covid in previously hospitalised children using the ISARIC global follow-up protocol: a prospective cohort study. Eur Respir J 2021:2101341. 10.1183/13993003.01341-2021 PMC8576804 34210789

[13] Sante GD , Buonsenso D , De Rose C , et al . Immune profile of children with post-acute sequelae of SARS-CoV-2 infection (long covid). Pediatrics [Preprint]. 10.1101/2021.05.07.21256539

[14] Miller F , Nguyen DV , Navaratnam AM , et al . Prevalence and characteristics of persistent symptoms in children during the COVID-19 pandemic: evidence from a household cohort study in England and Wales. Pediatr Infect Dis J 2022;41:979–84. 10.1097/INF.0000000000003715 36375098 PMC9645448

[15] Petersen MS , Kristiansen MF , Hanusson KD , et al . Long COVID in the faroe islands: a longitudinal study among nonhospitalized patients. Clin Infect Dis 2021;73:e4058–63. 10.1093/cid/ciaa1792 33252665 PMC7799340

[16] Office_for_National_Statistics . The prevalence of long COVID symptoms and COVID-19 complications 2020. Available: https://www.ons.gov.uk/news/statementsandletters/theprevalenceoflongcovidsymptomsandcovid19complications [Accessed 3 Aug 2020].

[17] Office_for_National_Statistics . Prevalence of ongoing symptoms following coronavirus (COVID-19) infection in the UK: 3 march 2022 edition of this dataset. 2022. Available: https://www.ons.gov.uk/peoplepopulationandcommunity/healthandsocialcare/conditionsanddiseases/datasets/alldatarelatingtoprevalenceofongoingsymptomsfollowingcoronaviruscovid19infectionintheuk [Accessed 3 Apr 2022].

[18] Riley S , Atchison C , Ashby D , et al . Real-time assessment of community transmission (react) of SARS-CoV-2 virus: study protocol. Wellcome Open Res 2020;5:200. 10.12688/wellcomeopenres.16228.2 33997297 PMC8095190

[19] Eales O , Wang H , Haw D , et al . Trends in SARS-CoV-2 infection prevalence during England’s roadmap out of lockdown, January to July 2021. PLoS Comput Biol 2022;18:e1010724. 10.1371/journal.pcbi.1010724 36417468 PMC9728904

[20] Imperial_College_London . Real-time assessment of community transmission (REACT) study: for researchers. Available: https://www.imperial.ac.uk/medicine/research-and-impact/groups/react-study/for-researchers [Accessed 11 Nov 2022].

[21] Office_for_National_Statistics . Comparisons of all-cause mortality between European countries and regions: January to June 2020. 2020. Available: https://www.ons.gov.uk/peoplepopulationandcommunity/birthsdeathsandmarriages/deaths/articles/comparisonsofallcausemortalitybetweeneuropeancountriesandregions/januarytojune2020 [Accessed 3 Apr 2022].

[22] Elliott P , Bodinier B , Eales O , et al . Rapid increase in omicron infections in England during December 2021: REACT-1 study. Science 2022;375:1406–11. 10.1126/science.abn8347 35133177 PMC8939772

[23] Riley S , Ainslie KEC , Eales O , et al . Resurgence of SARS-CoV-2: detection by community viral surveillance. Science 2021;372:990–5. 10.1126/science.abf0874 33893241 PMC8158959

[24] Pinto Pereira SM , Nugawela MD , Rojas NK , et al . Post-COVID-19 condition at 6 months and COVID-19 vaccination in non-hospitalised children and young people. Arch Dis Child 2023;108:289–95. 10.1136/archdischild-2022-324656 36599625 PMC10086284

[25] Grint DJ , Wing K , Houlihan C , et al . Severity of severe acute respiratory system coronavirus 2 (SARS-CoV-2) alpha variant (B.1.1.7) in England. Clin Infect Dis 2022;75:e1120–7. 10.1093/cid/ciab754 34487522 PMC8522415

[26] Stephenson T , Allin B , Nugawela MD , et al . Long COVID (post-COVID-19 condition) in children: a modified Delphi process. Arch Dis Child 2022;107:674–80. 10.1136/archdischild-2021-323624 35365499 PMC8983414

[27] Antonelli M , Pujol JC , Spector TD , et al . Risk of long COVID associated with delta versus omicron variants of SARS-cov-2. Lancet 2022;399:2263–4. 10.1016/S0140-6736(22)00941-2 35717982 PMC9212672

[28] UK_Government . GOV.UK coronavirus (COVID-19) in the UK 2022. Available: https://coronavirus.data.gov.uk/ [Accessed 18 Aug 2022].

