# Supplementary material for: Characteristics and predictors of persistent symptoms post-COVID-19 in children and young people: a large community cross-sectional study in England
Source: Arch Dis Child. 2023 Mar 2;108(7):e12. doi: 10.1136/archdischild-2022-325152 (PMC10313975; doi:10.1136/archdischild-2022-325152)
Supplement: Supplementary data [file archdischild-2022-325152supp001.pdf]

Supplementary File: Cluster analysis

Methods

We carried out a hierarchical cluster analysis to cluster individuals by reported symptoms at 3 months or more post COVID-19. First, an analysis using Ward's method applying squared Euclidean Distance as the distance or similarity measure was performed (1). This indicated the optimum number of clusters to work with. Second, the analysis was rerun with the selected number of clusters, which enabled every participant to be allocated to a particular cluster. Three scenarios of combinations of two, three and four clusters were used. The dendrogram of each hierarchical cluster analysis was evaluated to determine which symptom needed to be allocated to which cluster (Figure 1 & 2). The Calinski-Harabasz Index was used to select the optimal number of clusters (Table 1 & 2) (2). As sensitivity analysis, we also ran the hierarchical cluster analysis applying Jaccard Distance which unlike squared Euclidean Distance does not consider negative co-occurrence (3).

To compare the clusters by key participant characteristics (sex, ethnicity, presence of a pre-existing health condition and IMD) contingency tables and chi-squared tests (significance level: $p<0.05$) were used. To compare the clusters by age, ANOVA (significance level: $p<0.05$) was used. The prevalence of the symptoms in each constructed cluster was also estimated.

Results

Two clusters were identified in children aged 5-11 years based on symptom profiles at 3 months (Figure 1, Figure 3 & Table 1). Cluster 1 (n=90) was characterised by moderate prevalence of coughing which co-occurred with loss or change of sense of smell or taste. Cluster 2 (n=23) had a broader symptom profile with high prevalence of abdominal issues, mild fatigue, headaches, difficulty sleeping, achy or cramping muscles and joint pain (Figure 3). There was no difference in sociodemographic characteristics between the two clusters (Table 2). In children and young people aged 12-17 years, two symptom clusters were also identified (Figure 2, Figure 3 & Table 3). There was high prevalence of loss or change of sense of smell or taste in Cluster 1 (n=396). Cluster 2 (n=343) was characterised by multiple symptoms dominated by mild fatigue, headaches and shortness of breath (Figure 3). Cluster 2 contained a higher proportion of individuals who reported a pre-existing health condition (45.8% (95% CI 40.6-51.1) vs. 29.8% (95% CI 25.5-34.5) in Cluster 1; $p<0.001$) and who reported that their initial COVID-19 episode was when the wild-type strain was dominant (39.9% (95% CI 34.9-45.2) vs. 24.0% (95% CI 20.0-28.5) in Cluster 1; $p<0.001$). Conversely, Cluster 1 contained a higher proportion of individuals who reported that their initial COVID-19 episode was when the Delta strain was dominant (62.6% (95% CI 57.7-67.3) vs. 43.7% (95% CI 38.6-49.0) in Cluster 2; $p<0.001$) (Table 4).

In clustering sensitivity analysis, using hierarchical clustering with Jaccard Distance, we identified 3 and 2 clusters in children aged 5-11 and 12-17 years, respectively. For children aged 5-11 years, one cluster had a broad symptom profile dominated by abdominal issues, mild fatigue, and headache, and contained the same participants from Cluster 2 as in the main analysis (which also had a broad symptom profile with high prevalence of abdominal issues,

mild fatigue and headache), as well as some participants from Cluster 1. The other two clusters identified for this age group were small and were dominated by loss or change of sense of smell or taste in one and coughing in the other (Figure 4 & Table 5). For children aged 12-17 years, there was high prevalence of loss or change of sense of smell or taste in one cluster and a broader symptom profile in the other, which contained most of the same participants from Cluster 1 and Cluster 2 as in the main analysis, respectively (Figure 4 & Table 5).

Our identification of two symptom clusters at 3 months in both age group, albeit small numbers in each cluster particularly for younger children, suggests that long-term sequelae after COVID-19 may have distinct subgroups in children. A previous study in children aged 11-17 years using latent class analysis, found evidence of clustering in symptoms reported at 3 months, with two subgroups: one had very low prevalence of most symptoms, while the second subgroup was characterised by multiple symptoms dominated by tiredness, headache, shortness of breath and dizziness, not too dissimilar from Cluster 2 in older children in our study (4).

With regard to limitations of the clustering analysis, while this provides some insights into symptom clustering among children with persistent symptoms following COVID-19 our sample size was small, particularly for younger children. In addition, given the unpredictable emergence of new SARS-CoV-2 variants, it is unclear how stable this symptom clustering is over time.

Figure 1: Dendrogram of persistent symptom clusters among participants aged 5-11 years, N=113

Dendrograms graphically present the information concerning which observations (participants) are grouped together at various levels of (dis)similarity. At the bottom of the dendrogram, each observation is considered its own cluster. Vertical lines extend up for each observation, and at various (dis)similarity values, these lines are connected to the lines from other observations with a horizontal line. The observations continue to combine until, at the top of the dendrogram, all observations are grouped together. The height of the vertical lines and the range of the (dis)similarity axis give visual clues about the strength of the clustering. Long vertical lines indicate more distinct separation between the groups. Long vertical lines at the top of the dendrogram indicate that the groups represented by those lines are well separated from one another. Shorter lines indicate groups that are not as distinct.

We have limited our view to the top 10 branches of the dendrogram, labelled G1-10. The number of observations in each group is given below the label.

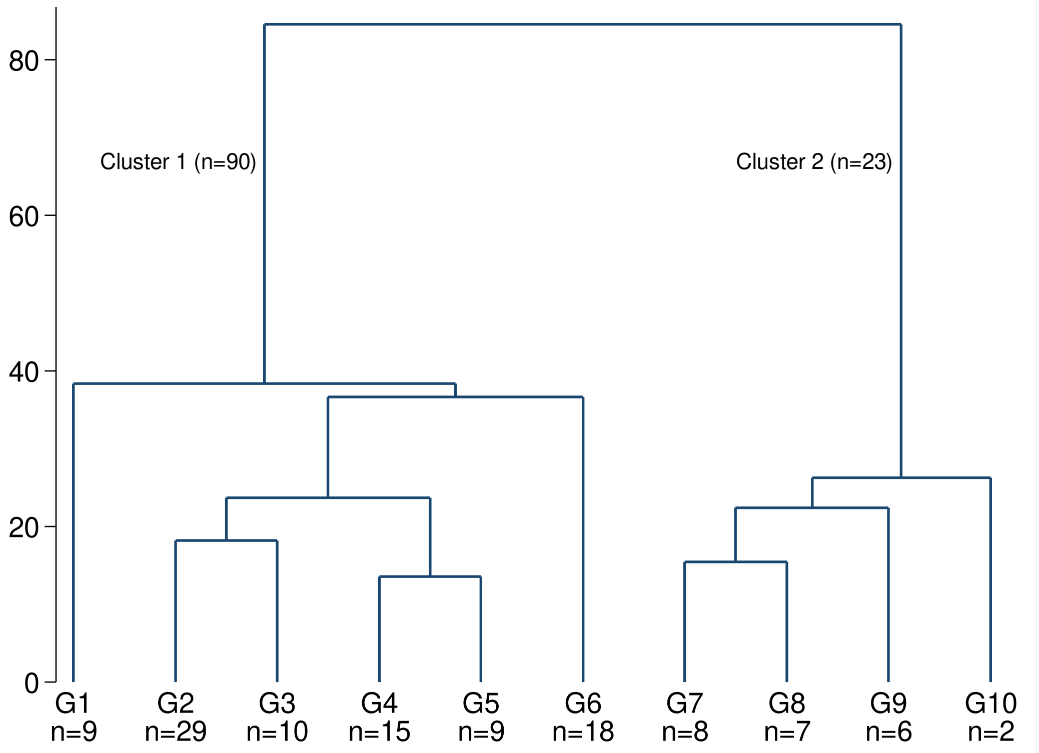

Table 1: Calinski-Harabasz Index for persistent symptom cluster analysis for participants aged 5-11 years, N=113

Calinski-Harabasz Index: Based on the idea that clusters that are 1) themselves very compact and 2) well-spaced from each other are good clusters. The index is calculated by dividing the variance of the sums of squares of the distances of individual objects to their cluster centre by the sum of squares of the distance between the cluster centers. Higher the Calinski-Harabasz Index, the better the clustering model. In this case the Calinski-Harabasz Index is maximised when the number of clusters is 2.

Number of clusters	Calinski-Harabasz Index
2	20.26
3	15.92
4	14.94
5	13.87
6	13.26
7	12.98
8	12.62
9	12.24
10	11.90
11	11.64
12	11.41
13	11.23
14	11.09
15	11.04

Table 2: Key characteristics of 3 month symptom clusters among participants aged 5-11 years with symptoms and date of symptom onset 3 months or more before survey date, N=113

For continuous variables, mean and standard deviation (SD) and analysis of variance (ANOVA) to test the difference between means. For categorical variables, column-wise within-group percentages shown in square brackets, with 95% confidence intervals in parentheses and chi-squared test to determine difference between frequencies in one or more categories.

	Category	Cluster 1 N=90 % (95% CI)	Cluster 2 N=23 % (95% CI)
**Age**	Mean (SD)	9.2 (1.7)	9.9 (1.3)
**Sex**	Male	39 [43.3 (33.4, 53.9)]	14 [60.9 (39.6, 78.7)]
	Female	51 [56.7 (46.1, 66.6)]	9 [39.1 (21.3, 60.4)]
**Ethnicity**	White	82 [91.1 (83.1, 95.5)]	19 [86.4 (64.2, 95.7)]
	Mixed	5 [5.6 (2.3, 12.8)]	2 [9.1 (2.2, 31.0)]
	Asian / Asian British	2 [2.2 (0.54, 8.6)]	0 [0.0 (0.0, 0.15)]
	Black / African / Caribbean / Black British	0 [0.0 (0.0, 0.04)]	1 [4.6 (0.59, 27.5)]
	Other	1 [1.1 (0.15, 7.7)]	0 [0.0 (0.0, 0.15)]
**Comorbidities**	No	68 [75.6 (65.5, 83.4)]	15 [65.2 (43.6, 82.0)]
	Yes	22 [24.4 (16.6, 34.5)]	8 [34.8 (18.0, 56.4)]
**IMD quintile**	1 – most deprived	4 [4.4 (1.7, 11.4)]	3 [13.0 (4.1, 34.5)]
	2	18 [20.0 (12.9, 29.7)]	2 [8.7 (2.1, 29.9)]
	3	14 [15.6 (9.4, 24.7)]	7 [30.4 (14.9, 52.3)]
	4	23 [25.6 (17.5, 35.7)]	6 [26.1 (11.9, 48.0)]
	5 – least deprived	31 [34.4 (25.2, 45.0)]	5 [21.7 (9.1, 43.6)]
**Dominant variant at time of infection**	Wild type (before Dec 2020)	42 [46.7 (36.5, 57.1)]	14 [60.9 (39.6, 78.7)]
	Alpha (Dec 2020-April 2021)	10 [11.1 (6.0, 19.6)]	2 [8.7 (2.1, 29.9)]
	Delta (May 2021-Dec 2021)	38 [42.2 (32.3, 52.8)]	7 [30.4 (14.9, 52.3)]

Figure 2: Dendrogram of persistent symptom clusters among participants aged 12-17 years, N=739

Dendrograms graphically present the information concerning which observations (participants) are grouped together at various levels of (dis)similarity. At the bottom of the dendrogram, each observation is considered its own cluster. Vertical lines extend up for each observation, and at various (dis)similarity values, these lines are connected to the lines from other observations with a horizontal line. The observations continue to combine until, at the top of the dendrogram, all observations are grouped together. The height of the vertical lines and the range of the (dis)similarity axis give visual clues about the strength of the clustering. Long vertical lines indicate more distinct separation between the groups. Long vertical lines at the top of the dendrogram indicate that the groups represented by those lines are well separated from one another. Shorter lines indicate groups that are not as distinct.

We have limited our view to the top 10 branches of the dendrogram, labelled G1-10. The number of observations in each group is given below the label.

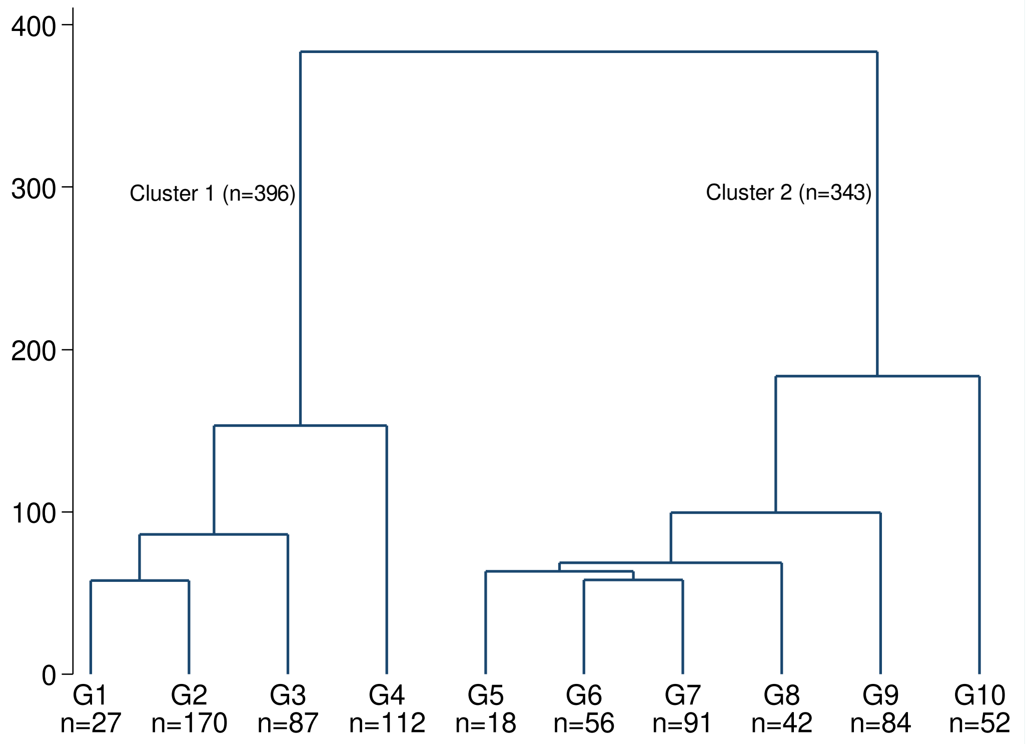

Table 3: Calinski-Harabasz Index for persistent symptom cluster analysis for participants aged 5-11 years, N=739

Calinski-Harabasz Index: Based on the idea that clusters that are 1) themselves very compact and 2) well-spaced from each other are good clusters. The index is calculated by dividing the variance of the sums of squares of the distances of individual objects to their cluster centre by the sum of squares of the distance between the cluster centers. Higher the Calinski-Harabasz Index, the better the clustering model. In this case the Calinski-Harabasz Index is maximised when the number of clusters is 2.

Number of clusters	Calinski-Harabasz Index
2	111.34
3	88.61
4	80.17
5	71.56
6	65.86
7	61.04
8	57.53
9	54.77
10	52.85
11	51.34
12	50.03
13	48.84
14	47.69
15	46.43

Table 4: Key characteristics of 3 month symptom clusters among participants aged 12-17 years with symptoms and date of symptom onset 3 months or more before survey date, N=739

For continuous variables, mean and standard deviation (SD) and analysis of variance (ANOVA) to test the difference between means. For categorical variables, column-wise within-group percentages shown in square brackets, with 95% confidence intervals in parentheses and chi-squared test to determine difference between frequencies in one or more categories. Cluster 2 contained significantly more individuals who had a pre-existing health condition (45.8% vs 29.8% in Cluster 1; p<0.001) and who had their initial COVID-19 episode when the Wild type strain was dominant (39.9% vs 24.0% in Cluster 1; p<0.001). Cluster 1 contained significantly more individuals who had their initial COVID-19 episode when the Delta strain was dominant (62.6% vs 43.7% in Cluster 2; p<0.001).

	**Category**	**Cluster 1 N=396 % (95% CI)**	**Cluster 2 N=343 % (95% CI)**
**Age**	Mean (SD)	15.0 (1.4)	15.0 (1.6)
**Sex**	Male	106 [26.8 (22.6, 31.4)]	78 [22.7 (18.6, 27.5)]
	Female	290 [73.2 (68.6, 77.4)]	265 [77.3 (72.5, 81.4)]
**Ethnicity**	White	349 [88.6 (85.0, 91.4)]	288 [84.5 (80.2, 87.9)]
	Mixed	16 [4.1 (2.5, 6.5)]	17 [5.0 (3.1, 7.9)]
	Asian / Asian British	21 [5.3 (3.5, 8.0)]	20 [5.9 (3.8, 8.9)]
	Black / African / Caribbean / Black British	5 [1.3 (0.53, 3.0)]	8 [2.4 (1.2, 4.6)]
	Other	3 [0.76 (0.24, 2.3)]	8 [2.4 (1.2, 4.6)]
**Comorbidities**	No	278 [70.2 (65.5, 74.5)]	186 [54.2 (48.9, 59.4)]
	Yes	118 [29.8 (25.5, 34.5)]	157 [45.8 (40.6, 51.1)]
**IMD quintile**	1 – most deprived	52 [13.1 (10.1, 16.8)]	53 [15.5 (12.0, 19.7)]
	2	59 [14.9 (11.7, 18.8)]	62 [18.1 (14.3, 22.5)]
	3	75 [18.9 (15.4, 23.1)]	59 [17.2 (13.6, 21.6)]
	4	97 [24.5 (20.5, 29.0)]	65 [19.0 (15.1, 23.5)]
	5 – least deprived	113 [28.5 (24.3, 33.2)]	104 [30.3 (25.7, 35.4)]
**Vaccination status at time of infection**	No	384 [97.0 (94.7, 98.3)]	323 [94.2 (91.1, 96.2)]
	At least one dose	12 [3.0 (1.7, 5.3)]	20 [5.8 (3.8, 8.9)]
**Dominant variant at time of infection**	Wild type (before Dec 2020)	95 [24.0 (20.0, 28.5)]	137 [39.9 (34.9, 45.2)]
	Alpha (Dec 2020-April 2021)	53 [13.4 (10.4, 17.1)]	56 [16.3 (12.8, 20.6)]
	Delta (May 2021-Dec 2021)	248 [62.6 (57.7, 67.3)]	150 [43.7 (38.6, 49.0)]

Figure 3: Results of clustering on symptom profile at 3 months or more post COVID-19 onset for participants aged 5-11 and 12-17 years, using hierarchical clustering. Darker red shading indicates higher symptom prevalence within the cluster. N=852

5-11 year olds			12-17 year olds	
**Cluster 1 (n=90)**	**Cluster 2 (n=23)**	**Symptoms at 3 months or more post COVID-19 onset**	**Cluster 1 (n=396)**	**Cluster 2 (n=343)**
6.7	82.6	Abdominal issues (stomach ache, diarrhoea, nausea, vomiting)	0.76	13.7
13.3	69.6	Mild fatigue (e.g. feeling tired)	7.3	32.1
14.4	65.2	Headaches	1	29.7
6.7	60.9	Difficulty sleeping	2	22.2
3.3	56.5	Achy or cramping muscles, pain in muscles	0.76	14.9
2.2	47.8	Pain in joints	1	12
3.3	34.8	Appetite loss	10.9	9.3
4.4	30.4	Confusion “brain fog”, forgetfulness	1	19.5
15.6	21.7	Loss or change of sense of taste	66.7	18.1
3.3	21.7	Tightness or heaviness in chest, chest pain	1.8	18.4
2.2	21.7	Weight loss	2.5	3.8
1.1	21.7	Heart issues (racing heart, palpitations, irregular heartbeat etc)	1	11.4
15.6	17.4	Loss or change of sense of smell	84.1	23.3
14.4	17.4	Shortness of breath, breathlessness, wheezing	3.3	28.9
4.4	13	Dizziness, vertigo	0.51	14.6
32.2	8.7	Coughing	1.3	10.8
7.8	8.7	Skin issues (itchy, scaly, redness, etc)	0	7.9
4.4	8.7	Itchy, sore or red eyes, conjunctivitis	0.25	2.9
2.2	8.7	Severe fatigue (e.g. inability to get out of bed)	0.51	11.1
0	8.7	Hair loss	1	6.7
10	4.4	Sneezing	0.25	5.3
7.8	4.4	Runny or blocked nose	1.5	10.5
6.7	4.4	Sore throat or hoarse voice	0.76	3.8
3.3	4.4	Fever	0.25	1.5
3.3	4.4	Hearing issues (e.g. hearing loss, Tinnitus etc)	0.51	3.2
0	4.4	Numbness or tingling somewhere in the body	1.5	2.6
4.4	0	Red/purple sores or blisters on your feet (including toes)	0.25	4.4
1.1	0	Vision issues	0	4.1
0	0	Sudden swelling of the face or lips	0	0
0	0	Leg swelling (Thrombosis)	0	0.29

Figure 4: Results of clustering (with different distance metric) on symptom profile at 3 months or more post COVID-19 onset for children aged 5-11 and 12-17 years, using the same hierarchical clustering approach as in the main analysis but replacing squared Euclidean Distance with Jaccard Distance, N=852

The plot shows cluster membership overlap between sensitivity analysis (Jaccard) and the main analysis (squared Euclidean Distance – Figure 3). For children aged 5-11 years, three clusters (Cluster S1-3) with Jaccard Distance created the best clusters according to the Calinski-Harabasz Index (Table 5). Cluster S1, with a broad symptom profile characterised by abdominal issues, mild fatigue and headache, included all the same observations from Cluster 2 from the main analysis (which also had a broad symptom profile with high prevalence of abdominal issues, mild fatigue and headache), as well as some observations from Cluster 1. Cluster S2 is a small group in which loss or change of sense of smell and taste are the predominant symptoms. Cluster 3 is a small group in which coughing is the only symptom. For children aged 12-17 years, two clusters (Cluster C1 and Cluster C2) with Jaccard Distance created the best clusters according to the Calinski-Harabasz Index (Table 5) which included most of the same observations from Cluster 1 and Cluster 2 from the main analysis, respectively. Similar to the main analysis, there was high prevalence of loss or change of sense of smell and taste in one cluster and a broader symptom profile in the other.

5-11 year olds				12-17 year olds	
Cluster S1 (n=84)	Cluster S2 (n=17)	Cluster S3 (n=12)	Symptoms at 3 months or more post COVID-19 onset	Cluster C1 (n=311)	Cluster C2 (n=428)
29.8	0	0	Abdominal issues (stomach ache, diarrhoea, nausea, vomiting)	0	11.7
33.3	0	0	Mild fatigue (e.g. feeling tired)	0	32.5
33.3	0	0	Headaches	0	24.8
23.8	0	0	Difficulty sleeping	0	19.6
19.1	0	0	Achy or cramping muscles, pain in muscles	0	12.6
15.5	0	0	Pain in joints	0	10.5
10.7	11.8	0	Appetite loss	0	17.5
13.1	0	0	Confusion “brain fog”, forgetfulness	0	16.6
7.1	76.5	0	Loss or change of sense of taste	64.0	29.7
9.5	0	0	Tightness or heaviness in chest, chest pain	0	16.4
7.1	5.9	0	Weight loss	0	5.4
7.1	0	0	Heart issues (racing heart, palpitations, irregular heartbeat etc)	0	10.1
7.1	70.6	0	Loss or change of sense of smell	86.5	33.6
20.2	0	0	Shortness of breath, breathlessness, wheezing	0	26.2
8.3	0	0	Dizziness, vertigo	0	12.2
22.6	0	100	Coughing	0	9.8
9.5	5.9	0	Skin issues (itchy, scaly, redness, etc)	0	6.3
6	5.9	0	Itchy, sore or red eyes, conjunctivitis	0	2.6
4.8	0	0	Severe fatigue (e.g. inability to get out of bed)	0	9.4
2.4	0	0	Hair loss	0	6.3
11.9	0	0	Sneezing	0	4.4
9.5	0	0	Runny or blocked nose	0	9.8
8.3	0	0	Sore throat or hoarse voice	0	3.7
4.8	0	0	Fever	0	1.4
4.8	0	0	Hearing issues (e.g. hearing loss, Tinnitus etc)	0	3.0
1.2	0	0	Numbness or tingling somewhere in the body	0	3.5
4.8	0	0	Red/purple sores or blisters on your feet (including toes)	0	3.7
1.2	0	0	Vision issues	0	3.3

0	0	0	Sudden swelling of the face or lips	0	0
0	0	0	Leg swelling (Thrombosis)	0	0.23

Table 5: Calinski-Harabasz Index for persistent symptom cluster analysis for participants aged 5-11 and 12-17 years, N=852

Calinski-Harabasz Index: Based on the idea that clusters that are 1) themselves very compact and 2) well-spaced from each other are good clusters. The index is calculated by dividing the variance of the sums of squares of the distances of individual objects to their cluster centre by the sum of squares of the distance between the cluster centers. Higher the Calinski-Harabasz Index, the better the clustering model. In this case the Calinski-Harabasz Index is maximised when the number of clusters is 3 and 2 for 5-11 year olds and 12-17 year olds, respectively.

Number of clusters	Calinski-Harabasz Index: 5-11 years old	Calinski-Harabasz Index: 12-17 years old
2	5.16	88.47
3	7.57	69.28
4	7.28	54.19
5	5.97	57.68
6	7.24	49.35
7	7.06	48.78
8	6.39	44.27
9	6.87	40.02
10	6.52	39.62
11	6.27	37.49
12	7.02	35.52
13	6.60	38.17
14	6.49	35.89
15	6.17	34.76

References

1. Souza-Dantas VC, Dal-Pizzol F, Tomasi CD, Spector N, Soares M, Bozza FA, et al. Identification of distinct clinical phenotypes in mechanically ventilated patients with acute brain dysfunction using cluster analysis. Medicine (Baltimore). 2020;99(18):e20041.
2. Caliński T, Harabasz J. A dendrite method for cluster analysis. Communications in Statistics. 1974;3(1):1-27.
3. Mainali KP, Slud E, Singer MC, Fagan WF. A better index for analysis of co-occurrence and similarity. Sci Adv. 2022;8(4):eabj9204.
4. Stephenson T, Pinto Pereira SM, Shafran R, de Stavola BL, Rojas N, McOwat K, et al. Physical and mental health 3 months after SARS-CoV-2 infection (long COVID) among adolescents in England (CLoCk): a national matched cohort study. Lancet Child Adolesc Health. 2022;6(4):230-9.
