# Supplementary material for: Characteristics and predictors of persistent symptoms post-COVID-19 in children and young people: a large community cross-sectional study in England
Source: Arch Dis Child. 2023 Mar 2;108(7):e12. doi: 10.1136/archdischild-2022-325152 (PMC10313975; doi:10.1136/archdischild-2022-325152)

Supplementary Figure S1. Study population flowchart

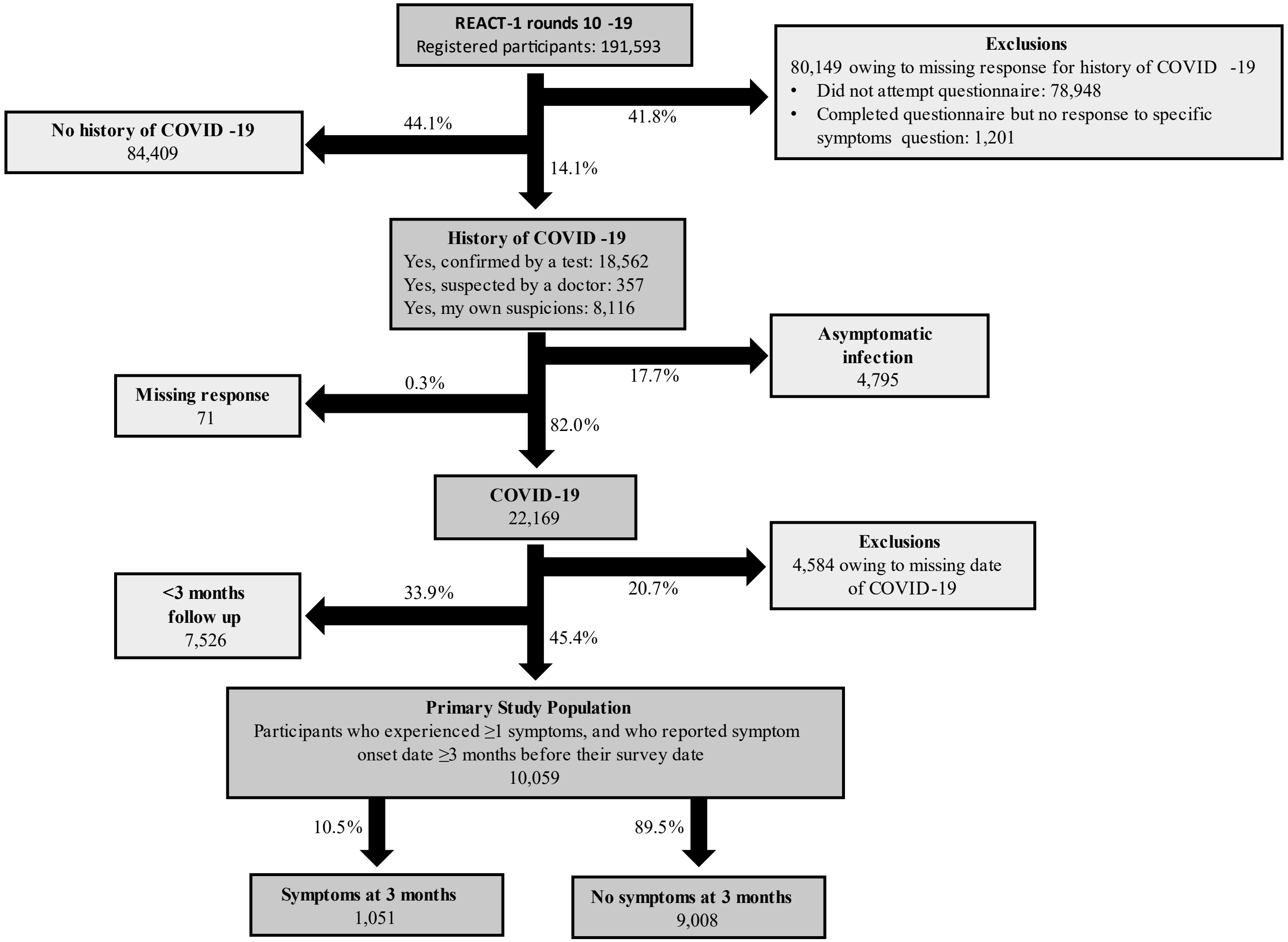

Supplementary Figure S2. Persistence of symptoms over time

Plot showing persistence of symptoms as a percentage of respondents who were symptomatic at time of infection and for whom we had 3 months follow-up and complete data, N=10,059 (*at least 6 months follow up data, N=463). Presented separately for participants aged 5-11 years (black) and 12-17 years (grey).

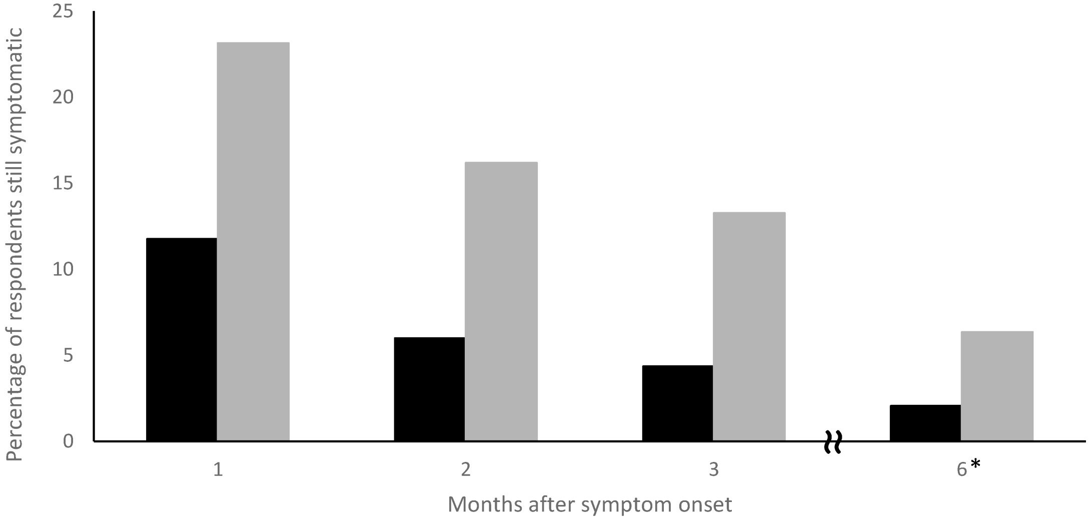

Supplementary Figure S3. Prevalence of symptoms persisting for 11 days or more in 123,468 PCR negative children aged 5-17 years in REACT-1.

Participants were asked about 26 separate symptoms lasting 11 days or more that were present within the 7 days of completing the questionnaire. The list of symptoms for this question was fewer than the list of 30 symptoms used for the question regarding persistent symptoms post COVID-19. Data collected between March 2021 and March 2022 (rounds 10-19). Error bars indicate 95% binomial confidence intervals of the prevalence. Average weighted prevalence of 11+ days persistent symptoms was 2.2% (95% CI 2.1–2.3) and 2.6% (95% CI 2.5–2.7) for children aged 5-11 and 12-17 years, respectively across rounds 10-19 of REACT-1.

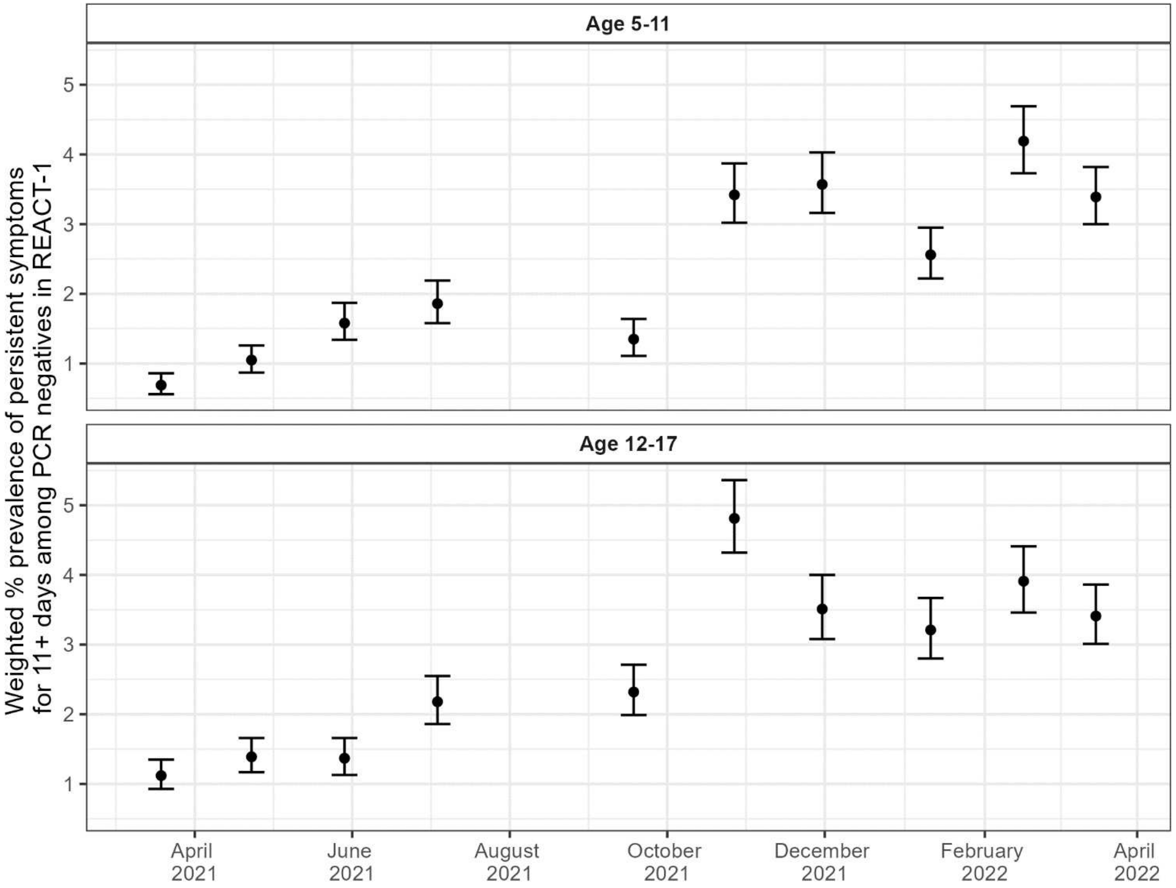

Supplement: Supplementary data [file archdischild-2022-325152supp002.pdf]
