# Supplementary material for: Characteristics and predictors of persistent symptoms post-COVID-19 in children and young people: a large community cross-sectional study in England
Source: Arch Dis Child. 2023 Mar 2;108(7):e12. doi: 10.1136/archdischild-2022-325152 (PMC10313975; doi:10.1136/archdischild-2022-325152)
Supplement: Supplementary data [file archdischild-2022-325152supp003.pdf]

Table S1: Key characteristics of the REACT-1 study population aged 5-17 years for rounds 10-19. Numbers are reported for the full sample and by history of COVID-19 status, N=191,593

	Category	Overall N (%; 95% CI)[a]	Response to history of COVID-19 N (%; 95% CI)[a]	Missing response to history of COVID-19 N (%; 95% CI)[a]	p-value [b]
	All participants	191,593	111,444 (58.2; 57.9-58.4)	80,149 (41.8; 41.6-42.1)	
**Sex**	Male	91,788 (47.9; 47.7-48.1)	52,190 (46.8; 46.5-47.1)	39,598 (49.4; 49.1-49.8)	<0.001
	Female	99,805 (52.1; 51.9-52.3)	59,254 (53.2; 52.9-53.5)	40,551 (50.6; 50.2-50.9)	
**Age**	5-11	87,915 (45.9; 45.7-46.1)	50,438 (45.3; 45.0-45.6)	37,477 (46.8; 46.4-47.1)	<0.001
	12-17	103,678 (54.1; 53.9-54.3)	61,006 (54.7; 54.4-55.0)	42,672 (53.2; 52.9-53.6)	
**Ethnicity**	White	146,882 (78.4; 78.2-78.6)	88,090 (80.5; 80.3-80.7)	58,792 (75.5; 75.2-75.8)	<0.001
	Mixed	10,760 (5.7; 5.6-5.9)	6,387 (5.8; 5.7-6.0)	4,373 (5.6; 5.5-5.8)	
	Asian / Asian British	19,595 (10.5; 10.3-10.6)	10,004 (9.1; 9.0-9.3)	9,591 (12.3; 12.1-12.5)	
	Black / African / Caribbean / Black British	7,085 (3.8; 3.7-3.9)	3,435 (3.1; 3.0-3.2)	3,650 (4.7; 4.5-4.8)	
	Other	2,980 (1.6; 1.5-1.7)	1,503 (1.4; 1.3-1.4)	1,477 (1.90 1.4-1.5)	
**IMD quintile**	1 – most deprived	28,810 (15.0; 14.9-15.2)	14,098 (12.7; 12.5-12.8)	14,712 (18.4; 18.1-18.6)	<0.001
	2	32,572 (17.0; 16.8-17.2)	17,624 (15.8; 15.6-16.0)	14,948 (18.7; 18.4-18.9)	
	3	37,837 (19.7; 19.6-19.9)	22,181 (19.9; 19.7-20.1)	15,656 (19.5; 19.3-19.8)	
	4	41,725 (21.8; 21.6-22.0)	25,443 (22.8; 22.6-23.1)	16,282 (20.3; 20.0-20.6)	
	5 – least deprived	50,649 (26.4; 26.2-26.6)	32,098 (28.8; 28.5-29.1)	18,551 (23.2; 22.9-23.4)	
**Region**	North East	7,510 (3.92; 3.83-4.01)	4,146 (3.72; 3.61-3.83)	3,364 (4.20; 4.06-4.34)	<0.001
	North West	21,715 (11.3; 11.2-11.5)	12,067 (10.8; 10.6-11.0)	9,648 (12.0; 11.8-12.3)	
	Yorkshire and The Humber	16,992 (8.87; 8.74-9.00)	9,760 (8.76; 8.59-8.93)	7,232 (9.02; 8.83-9.22)	
	East Midlands	18,415 (9.61; 9.48-9.74)	10,685 (9.59; 9.42-9.76)	7,730 (9.64; 9.44-9.85)	
	West Midlands	19,746 (10.3; 10.2-10.4)	11,023 (9.89; 9.72-10.1)	8,723 (10.9; 10.7-11.1)	
	East of England	23,169 (12.1; 11.9-12.2)	13,841 (12.4; 12.2-12.6)	9,328 (11.6; 11.4-11.9)	

	London	29,765 (15.5; 15.4-15.7)	17,004 (15.3; 15.0-15.5)	12,761 (15.9; 15.7-16.2)	
	South East	35,683 (18.6; 18.5-18.8)	21,595 (19.4; 19.1-19.6)	14,088 (17.6; 17.3-17.8)	
	South West	18,598 (9.71; 9.58-9.84)	11,323 (10.2; 9.98-10.3)	7,275 (9.08; 8.88-9.28)	
**Round**	10	25,049 (13.1; 12.9-13.2)	15,955 (14.3; 14.1-14.5)	9,094 (11.4; 11.1-11.6)	<0.001
	11	21,625 (11.3; 11.1-11.4)	13,269 (11.9; 11.7-12.1)	8,356 (10.4; 10.2-10.6)	
	12	21,795 (11.4; 11.2-11.5)	11,432 (10.3; 10.1-10.4)	10,363 (12.9; 12.7-13.2)	
	13	19,773 (10.3; 10.2-10.5)	10,542 (9.46; 9.29-9.63)	9,231 (11.5; 11.3-11.7)	
	14	18,393 (9.60; 9.47-9.73)	10,050 (9.02; 8.85-9.19)	8,343 (10.4; 10.2-10.6)	
	15	17,496 (9.13; 9.00-9.26)	10,175 (9.13; 8.96-9.30)	7,321 (9.13; 8.94-9.34)	
	16	15,391 (8.03; 7.91-8.16)	8,805 (7.90; 7.74-8.06)	6,586 (8.22; 8.03-8.41)	
	17	16,260 (8.49; 8.36-8.61)	9,838 (8.83; 8.66-9.00)	6,422 (8.01; 7.83-8.20)	
	18	16,716 (8.72; 8.60-8.85)	9,703 (8.71; 8.54-8.87)	7,013 (8.75; 8.56-8.95)	
	19	19,095 (9.97; 9.83-10.1)	11,675 (10.5; 10.3-10.7)	7,420 (9.26; 9.06-9.46)	

[a] Percentages are calculated from non-missing values; [b] P-value calculated using Pearson's chi-squared test

Table S2: Odds ratios for persistent symptoms at 3 months among symptomatic respondents, derived from logistic regression models used in the main analysis, N=10,059 (forest plot in Figure 2).

	Participants aged 5-11 years		Participants aged 12-17 years	
**Predictor**	**Crude (univariate) Odds Ratio (95% CIs)**	**[a]Adjusted Odds Ratio (95% CIs)**	**Crude (univariate) Odds Ratio (95% CIs)**	**[b]Adjusted Odds Ratio (95% CIs)**
**Sex**				
Male	-	-	-	-
Female	1.12 (0.80-1.58)	1.16 (0.82-1.64)	2.61 (2.22-3.06) ***	2.40 (2.03-2.82)***
**Ethnicity**				
White	-	-	-	-
Mixed	0.71 (0.36-1.42)	0.69 (0.34-1.38)	0.81 (0.58-1.13)	0.82 (0.58-1.15)
Asian / Asian British	0.32 (0.10-1.03)	0.35 (0.11-1.12)	0.67 (0.50-0.90)**	0.65 (0.48-0.88)**
Black / African / Caribbean / Black British	0.51 (0.07-3.71)	0.56 (0.08-4.16)	0.78 (0.45-1.37)	0.67 (0.37-1.18)
Other	0.59 (0.08-4.38)	0.63 (0.08-4.70)	1.54 (0.89-2.68)	1.26 (0.71-2.23)
**IMD quintile**				
1 – most deprived	-	-	-	-
2	1.66 (0.79-3.48)	1.70 (0.80-3.61)	0.97 (0.76-1.24)	0.96 (0.75-1.24)
3	1.21 (0.58-2.56)	1.17 (0.55-2.49)	0.75 (0.59-0.95)*	0.78 (0.61-0.99)*
4	1.44 (0.70-2.95)	1.39 (0.67-2.89)	0.72 (0.57-0.91)**	0.71 (0.55-0.91)**
5 – least deprived	1.38 (0.68-2.78)	1.34 (0.66-2.73)	0.76 (0.61-0.95)*	0.78 (0.62-0.98)*
**Comorbidities**				
No	-	-	-	-
Yes	2.59 (1.77-3.78)***	2.64 (1.80-3.90)***	2.17 (1.88-2.51)***	1.96 (1.68-2.27)***
**Vaccination status at time of infection**				
No	-	-	-	-
At least one dose	-	-	0.92 (0.66-1.28)	0.74 (0.52-1.04)
**Dominant variant at time of infection**				
Wild type (before Dec 2020)	-		-	-
Alpha (Dec 2020-April 2021)	0.85 (0.49-1.47)	0.92 (0.53-1.62)	1.18 (0.95-1.46)	1.16 (0.93-1.45)
Delta (May 2021-Dec 2021)	1.31 (0.91-1.89)	1.35 (0.93-1.97)	1.43 (1.23-1.67)***	1.56 (1.33-1.84)***

[a]Mutually adjusted for sex, ethnicity, IMD, comorbidities and dominant variant at time of infection; [b]Mutually adjusted for sex, ethnicity, IMD, comorbidities and vaccination status and dominant variant at time of infection; *p<0.05, *p<0.01*; ***p<0.001

Sensitivity analysis was performed restricting the outcome to test confirmed COVID-19.

In sensitivity analysis, restricting to children and young people who reported having had COVID-19 confirmed by a test, the magnitude and direction of associations between sociodemographic factors and persistent symptoms were similar (Supplementary Table S6).

Table S3. Number and proportion of individual symptoms among participants with persistent symptoms at 3 months or more post COVID-19 onset for whom we have 3 months follow-up and complete data.

	**Age 5-11**	**Age 12-17**
Symptom	n (%; 95% CI)	n (%; 95% CI)
Cohort with persistent symptoms at 3 months post COVID-19 onset / Cohort who experienced ≥1 symptom at time of infection, and who reported symptom onset date ≥3 months (≥91 days) before their survey date	138/3,173 (4.4%; 3.7-5.1)	913/6,886 (13.3%; 12.5-14.1)
Loss or change of sense of smell	19 (13.9; 9.0-20.8)	470 (52.2; 48.9-55.5)
Loss or change of sense of taste	21 (15.4; 10.3-22.6)	366 (40.7; 37.5-44.0)
Fever	4 (3.0; 1.1-7.7)	9 (1.0; 0.52-1.9)
Coughing	37 (27.4; 20.5-35.6)	57 (6.4; 4.9-8.1)
Runny or blocked nose	9 (6.7; 3.5-12.4)	49 (5.5; 4.2-7.2)
Sneezing	10 (7.3; 4.0-13.1)	23 (2.6; 1.7-3.8)
Itchy, sore or red eyes, conjunctivitis	7 (5.1; 2.4-10.4)	17 (1.9; 1.2-3.0)
Vision issues	1 (0.74; 0.10-5.1)	21 (2.3; 1.5-3.5)
Sore throat or hoarse voice	11 (8.0; 4.5-14.0)	28 (3.1; 2.2-4.5)
Hearing issues (e.g. hearing loss, Tinnitus etc)	4 (2.9; 1.1-7.5)	17 (1.9; 1.2-3.0)
Skin issues (itchy, scaly, redness, etc)	11 (8.0; 4.5-13.9)	33 (3.7; 2.6-5.1)
Sudden swelling of the face or lips	0 (0.0; 0.0-0.03)	0 (0.0, 0.0-0.01)
Leg swelling (Thrombosis)	0 (0.0; 0.0-0.03)	1 (0.11; 0.02-0.78)
Red/purple sores or blisters on your feet (including toes)	5 (3.6; 1.5-8.4)	20 (2.2; 1.4-3.4)
Hair loss	5 (3.6; 1.5-8.4)	34 (3.7; 2.7-5.2)
Appetite loss	15 (11.0; 6.7-17.5)	105 (11.8; 9.8-14.0)
Weight loss	7 (5.2; 2.5-10.4)	32 (3.5; 2.5-5.0)
Abdominal issues (stomach ache, diarrhoea, nausea, vomiting)	29 (21.8; 15.6-30.0)	77 (8.6; 6.9-10.6)
Dizziness, vertigo	8 (5.8; 2.9-11.3)	84 (9.4; 7.6-11.5)
Confusion "brain fog", forgetfulness	16 (11.6; 7.2-18.1)	92 (10.5; 8.6-12.7)
Headaches	33 (25.4; 18.6-33.6)	139 (16.3; 13.9-18.9)
Shortness of breath, breathlessness, wheezing	23 (16.7; 11.3-23.9)	149 (16.7; 14.4-19.3)
Tightness or heaviness in chest, chest pain	14 (10.2; 6.1-16.6)	106 (11.8; 9.9-14.1)
Heart issues (racing heart, palpitations, irregular heartbeat etc)	8 (5.8; 2.9-11.3)	63 (7.0; 5.5-8.8)

Difficulty sleeping	23 (16.7; 11.3-23.9)	127 (14.2; 12.0-16.6)
Mild fatigue (e.g. feeling tired)	34 (24.8; 18.3-32.8)	199 (22.1; 19.5-25.0)
Severe fatigue (e.g. inability to get out of bed)	5 (3.6; 1.5-8.4)	59 (6.6; 5.1-8.4)
Numbness or tingling somewhere in the body	1 (0.73; 0.10-5.0)	20 (2.2; 1.4-3.4)
Achy or cramping muscles, pain in muscles	21 (15.6; 10.3-22.7)	73 (8.1; 6.5-10.1)
Pain in joints	18 (13.1; 8.4-19.9)	61 (6.8; 5.3-8.6)

Table S4. Sociodemographic proportions of the REACT-1 study population aged 5-17 years for rounds 10-19 and participants included in our study compared to England population aged 5-17 years.

	England (5-17 population)[1, 2]	REACT-1 population (5-17)	Study population (5-17)
	N=8,853,841	N=191,593	N=111,444
	%	%	%
**Sex**			
Male	51.3	47.9	46.8
Female	48.7	52.1	53.2
**Age**			
5-11	55.7	45.9	45.3
12-17	44.3	54.1	54.7
**Ethnicity**			
White	79.2	78.4	80.5
Mixed	4.6	5.7	5.8
Asian	10.2	10.5	9.1
Black	4.6	3.8	3.1
Other	1.3	1.6	1.4
**Region**			
North East	4.5	3.9	3.7
North West	13.0	11.3	10.8
Yorkshire and The Humber	9.8	8.9	8.8
East Midlands	8.4	9.6	9.6
West Midlands	10.8	10.3	9.9
East of England	11.2	12.1	12.4
London	16.4	15.5	15.3
South East	16.6	18.6	19.4
South West	9.4	9.7	10.2
**IMD Quintile**			
1 – most deprived	19.8	15.0	12.7
2	20.9	17.0	15.8
3	20.4	19.7	19.9
4	19.7	21.8	22.8
5 – least deprived	19.1	26.4	28.8

1 Office_for_National_Statistics. Population estimates for the UK, England and Wales, Scotland and Northern Ireland: mid2019 2020 [Available from: https://www.ons.gov.uk/releases/populationestimatesfortheukenglandandwalesscotlandandnorthernirelandmid2019(

2 Office_for_National_Statistics. Employee earnings in the UK: 2019 2019 [Available from: https://www.ons.gov.uk/releases/employeeearningsintheuk2019.

Table S5. Number and proportion of individual symptoms among participants with persistent symptoms at 3 months or more post COVID-19 onset for whom we have 3 months follow-up and complete data, test confirmed vs. suspected COVID-19 cases.

	**Age 5-11**		**Age 12-17**	
Symptom	n (%; 95% CI)		n (%; 95% CI)	
	Test confirmed COVID-19	Suspected COVID-19	Test confirmed COVID-19	Suspected COVID-19
Cohort with persistent symptoms at 3 months post COVID-19 onset / Cohort who experienced ≥1 symptom at time of infection, and who reported symptom onset date ≥3 months (≥91 days) before their survey date	71/1,382 (5.1%; 4.1-6.4)	67/1,791 (3.7%; 3.0-4.7)	692/4,313 (16.0%; 15.0-17.2)	221/2,573 (8.6%; 7.6-9.7)
Loss or change of sense of smell	15 (21.4; 13.3-32.7)	4 (6.0; 2.2-15.0)	400 (58.7; 54.9-62.3)	70 (32.1; 26.2-38.6)
Loss or change of sense of taste	17 (24.3; 15.6-35.7)	4 (6.0; 2.2-15.0)	306 (45.0; 41.3-48.8)	60 (27.4; 21.9-33.7)
Fever	2 (2.9; 0.72-11.0)	2 (3.0; 0.75-11.4)	6 (0.87; 0.39-1.9)	3 (1.4; 0.45-4.3)
Coughing	15 (21.4; 13.3-32.7)	22 (33.8; 23.4-46.2)	36 (5.3; 3.8-7.2)	21 (9.6; 6.4-14.3)
Runny or blocked nose	6 (8.6; 3.9-17.9)	3 (4.5; 1.4-13.1)	40 (5.9; 4.4-8.0)	9 (4.2; 2.2-8.0)
Sneezing	6 (8.6; 3.9-17.9)	4 (6.0; 2.2-15.0)	19 (2.8; 1.8-4.3)	4 (1.8; 0.68-4.7)
Itchy, sore or red eyes, conjunctivitis	5 (7.1; 3.0-16.1)	2 (3.0; 0.75-11.4)	11 (1.6; 0.89-2.9)	6 (2.8; 1.2-6.0)
Vision issues	1 (1.4; 0.20-9.6)	0 (0.0; 0.0-0.61)	12 (1.7; 0.99-3.0)	9 (4.1; 2.1-7.7)
Sore throat or hoarse voice	8 (11.4; 5.8-21.3)	3 (4.5; 1.4-13.1)	17 (2.5; 1.6-4.0)	11 (5.0; 2.8-8.9)
Hearing issues (e.g. hearing loss, Tinnitus etc)	1 (1.4; 0.20-9.6)	3 (4.5; 1.4-13.1)	13 (1.9; 1.1-3.2)	4 (1.8; 0.68-4.7)
Skin issues (itchy, scaly, redness, etc)	4 (5.6; 2.1-14.2)	7 (10.4; 5.0-20.4)	20 (2.9; 1.9-4.5)	13 (6.0; 3.5-10.1)
Sudden swelling of the face or lips	0 (0.0; 0.0-0.57)	0 (0.0; 0.0-0.61)	0 (0.0, 0.0-0.11)	0 (0.0, 0.0-0.31)
Leg swelling (Thrombosis)	0 (0.0; 0.0-0.57)	0 (0.0; 0.0-0.61)	0 (0.0, 0.0-0.11)	1 (0.45; 0.06-3.2)
Red/purple sores or blisters on your feet (including toes)	1 (1.4; 0.20-9.6)	4 (6.0; 2.2-15.0)	3 (0.43; 0.14-1.3)	17 (7.7; 4.8-12.0)
Hair loss	3 (4.2; 1.4-12.4)	2 (3.0; 0.75-11.4)	24 (3.5; 2.3-5.2)	10 (4.5; 2.5-8.3)
Appetite loss	8 (11.4; 5.8-21.3)	7 (10.4; 5.0-20.4)	83 (12.2; 9.9-14.8)	22 (10.4; 7.0-15.3)
Weight loss	2 (2.9; 0.72-11.0)	5 (7.5; 3.1-16.8)	23 (3.4; 2.2-5.0)	9 (4.1; 2.1-7.7)
Abdominal issues (stomach ache, diarrhoea, nausea, vomiting)	14 (20.3; 12.3-31.5)	15 (23.4; 14.6-35.4)	46 (6.7; 5.1-8.9)	31 (14.6; 10.4-20.1)
Dizziness, vertigo	4 (5.6; 2.1-14.2)	4 (6.0; 2.2-15.0)	54 (7.9; 6.1-10.2)	30 (14.2; 10.1-19.5)
Confusion “brain fog”, forgetfulness	10 (14.1; 7.7-24.3)	6 (9.0; 4.1-18.6)	71 (10.6; 8.5-13.2)	21 (9.6; 6.4-14.3)
Headaches	19 (29.7; 19.7-42.0)	14 (20.9; 12.7-32.4)	102 (15.6; 13.0-18.6)	37 (18.5; 13.7-24.5)
Shortness of breath, breathlessness, wheezing	10 (14.1; 7.7-24.3)	13 (19.4; 11.6-30.7)	104 (15.4; 12.8-18.3)	45 (20.7; 15.8-26.7)
Tightness or heaviness in chest, chest pain	3 (4.2; 1.4-12.4)	11 (16.7; 9.4-27.8)	74 (10.8; 8.7-13.4)	32 (15.0; 10.8-20.5)
Heart issues (racing heart, palpitations, irregular heartbeat etc)	3 (4.2; 1.4-12.4)	5 (7.5; 3.1-16.8)	42 (6.1; 4.5-8.1)	21 (9.6; 6.4-14.3)
Difficulty sleeping	13 (18.3; 10.9-29.1)	10 (14.9; 8.2-25.7)	85 (12.5; 10.2-15.2)	42 (19.3; 14.6-25.1)
Mild fatigue (e.g. feeling tired)	20 (28.6; 19.2-40.3)	14 (20.9; 12.7-32.4)	151 (22.1; 19.2-25.4)	48 (22.1; 17.1-28.1)
Severe fatigue (e.g. inability to get out of bed)	3 (4.2; 1.4-12.4)	2 (3.0; 0.75-11.4)	42 (6.1; 4.5-8.1)	17 (7.7; 4.8-12.0)

Numbness or tingling somewhere in the body	0 (0.0; 0.0-0.57)	1 (1.5; 0.21-10.0)	13 (1.9; 1.1-3.2)	7 (3.2; 1.5-6.6)
Achy or cramping muscles, pain in muscles	10 (14.1; 7.7-24.3)	11 (16.7; 9.4-27.8)	52 (7.6; 5.8-9.9)	21 (9.6; 6.4-14.3)
Pain in joints	8 (11.4; 5.8-21.3)	10 (14.9; 8.2-25.7)	42 (6.1; 4.5-8.1)	19 (8.9; 5.7-13.5)

Table S6: Odds ratios for persistent symptoms at 3 months among symptomatic respondents, derived from logistic regression models used in sensitivity analysis, test confirmed COVID-19 cases only (N=5,695)

	Participants aged 5-11 years		Participants aged 12-17 years	
**Predictor**	**Crude (univariate) Odds Ratio (95% CIs)**	[a]**Adjusted Odds Ratio (95% CIs)**	**Crude (univariate) Odds Ratio (95% CIs)**	[b]**Adjusted Odds Ratio (95% CIs)**
**Sex**				
Male	-	-	-	-
Female	0.97 (0.60-1.57)	0.98 (0.60-1.61)	2.85 (2.35-3.45) ***	2.63 (2.17-3.20)***
**Ethnicity**				
White	-	-	-	-
Mixed	0.72 (0.26-2.04)	0.71 (0.25-2.01)	0.87 (0.60-1.27)	0.86 (0.58-1.27)
Asian / Asian British	0.33 (0.08-1.36)	0.36 (0.09-1.53)	0.64 (0.46-0.89)**	0.64 (0.46-0.91)*
Black / African / Caribbean / Black British	1.00 (0.13-7.65)	1.13 (0.14-8.83)	0.97 (0.50-1.86)	0.89 (0.45-1.74)
Other	1.42 (0.18-11.09)	1.29 (0.15-10.73)	1.67 (0.89-3.15)	1.36 (0.70-2.64)
**IMD quintile**				
1 – most deprived	-	-	-	-
2	1.56 (0.62-3.97)	1.56 (0.60-4.11)	1.00 (0.75-1.33)	1.03 (0.76-1.39)
3	0.85 (0.32-2.27)	0.92 (0.34-2.52)	0.82 (0.62-1.09)	0.86 (0.64-1.15)
4	0.86 (0.34-2.20)	0.88 (0.34-2.31)	0.81 (0.61-1.06)	0.84 (0.63-1.12)
5 – least deprived	1.51 (0.64-3.54)	1.58 (0.65-3.82)	0.87 (0.67-1.13)	0.93 (0.71-1.23)
**Comorbidities**				
No	-	-	-	-
Yes	2.35 (1.36-4.07)**	2.26 (1.28-4.00)**	2.36 (1.98-2.80)***	2.09 (1.75-2.49)***
**Vaccination status at time of infection**				
No	-	-	-	-
At least one dose	-	-	0.73 (0.52-1.03)	0.72 (0.50-1.03)
**Dominant variant at time of infection**				
Wild type (before Dec 2020)	-		-	-
Alpha (Dec 2020-April 2021)	0.78 (0.34-1.77)	0.75 (0.32-1.74)	0.64 (0.48-0.85)**	0.63 (0.47-0.84)**
Delta (May 2021-Dec 2021)	0.91 (0.46-1.78)	0.82 (0.41-1.66)	0.70 (0.56-0.87)**	0.75 (0.60-0.95)*

[a]Mutually adjusted for sex, ethnicity, IMD, comorbidities and dominant variant at time of infection; [b]Mutually adjusted for sex, ethnicity, IMD, comorbidities and vaccination status and dominant variant at time of infection; *p<0.05, *p<0.01*; ***p<0.001
